# Supplementary material for: A Likelihood Approach for Real-Time Calibration of Stochastic Compartmental Epidemic Models
Source: PLoS Comput Biol. 2017 Jan 17;13(1):e1005257. doi: 10.1371/journal.pcbi.1005257 (PMC5240920; doi:10.1371/journal.pcbi.1005257)
Supplement: S1 File — (TAR.GZ) [file pcbi.1005257.s014.tar.gz › HSPH_Online-SI-Revision/Additions/MSS11/n100-extreme-SEITR/n100-extreme-SEITR_NEW_table4.pdf]

1	R0	{1.38654, 9.85565, 1.}	{2.96797, 18.4042, 0.}	{{2.96797, 18.4042, 0.}, {2.96797, 18.4042, 0.}}	{114.056, 86.7376, 100.}	inf	4758.	8166.1	{8134.27, 8194.52}	71.6289	0.234588	newly	2747.	2678.4	{2625., 2731.}	2.57153	0.0113569	Reff	0.128602	0.191995	{0.190919, 0.19292}	49.2945	ar	92 379.	95 153.8	{95 089., 95 216.}	3.00371
2	R0	{2.36629, 12.2029, 0.1}	{2.9751, 8.95585, 1.}	{{2.9751, 8.95585, 1.}, {2.9751, 8.95585, 1.}}	{25.7283, 26.6088, 900.}	inf	5892.	1473.38	{1442.7, 1501.81}	74.9935	0.601992	newly	2664.	846.57	{816., 880.}	68.2218	0.49804	Reff	0.0301229	0.172425	{0.171567, 0.173331}	472.406	ar	99 022.	94 497.4	{94 452., 94 539.}	4.56927
3	R0	{2.32253, 12.836, 1.}	{2.99092, 10.3743, 0.}	{{2.99092, 10.3743, 0.}, {2.99092, 10.3743, 0.}}	{28.7784, 19.178, 100.}	inf	185.	2939.05	{2903.37, 2968.56}	62.4355	0.42524	newly	3413.	1528.32	{1489., 1568.}	55.2206	0.349012	Reff	0.035767	0.197698	{0.196699, 0.198713}	452.74	ar	98 997.	94 062.4	{94 009., 94 110.}	4.98465
4	R0	{1.79075, 11.2058, 1.}	{2.9884, 12.8326, 0.}	{{2.9884, 12.8326, 0.}, {2.9884, 12.8326, 0.}}	{66.8796, 14.5175, 100.}	inf	424.	3814.78	{3779.27, 3851.62}	43.1732	0.245458	newly	3304.	1694.07	{1654., 1738.}	48.7267	0.290202	Reff	0.0739401	0.222672	{0.221528, 0.223705}	201.152	ar	96 920.	93 543.3	{93 487., 93 599.}	3.48405
5	R0	{2.26952, 9.62371, 0.1}	{2.99949, 5.42517, 0.}	{{2.99949, 5.42517, 0.}, {2.99949, 5.42517, 0.}}	{32.1643, 43.6271, 100.}	inf	39.	10.3396	{2.65874, 19.4469}	99.5831	2.47222	newly	1318.	8.03	{2., 15.}	99.3907	2.32701	Reff	0.0289591	0.113521	{0.113246, 0.113743}	292.004	ar	98 881.	96 216.7	{96 208., 96 227.}	2.69448
6	R0	{1.57191, 6.89004, 0.1}	{2.97612, 8.62388, 4.}	{{2.97612, 8.62388, 4.}, {2.97612, 8.62388, 4.}}	{89.3314, 25.1644, 3900.}	inf	100.	382.106	{361.783, 406.086}	73.666	0.57999	newly	1057.	226.79	{208., 245.}	78.544	0.669455	Reff	0.0825567	0.191093	{0.190282, 0.19169}	131.469	ar	95 046.	93 666.2	{93 636., 93 700.}	1.45173
7	R0	{1.99226, 17.2339, 1.}	{2.96797, 18.4042, 0.}	{{2.96797, 18.4042, 0.}, {2.96797, 18.4042, 0.}}	{48.9752, 6.79072, 100.}	inf	602.	9231.87	{9198.77, 9270.16}	19.5549	0.0945029	newly	3977.	3015.91	{2961., 3067.}	24.1662	0.120178	Reff	0.0686533	0.186749	{0.185597, 0.187708}	172.017	ar	97 992.	95 457.2	{95 389., 95 535.}	2.58671
8	R0	{2.63369, 15.1879, 0.1}	{2.93906, 10.3908, 0.}	{{2.93906, 10.3908, 0.}, {2.93906, 10.3908, 0.}}	{11.5946, 31.5851, 100.}	inf	100.	2743.48	{2706.64, 2777.43}	66.5551	0.475694	newly	3068.	1423.05	{1390., 1458.}	53.6164	0.333716	Reff	0.022123	0.188085	{0.187051, 0.189136}	750.18	ar	99 451.	94 196.2	{94 134., 94 253.}	5.28379
9	R0	{2.53271, 9.90552, 0.1}	{2.99949, 5.42517, 0.}	{{2.99949, 5.42517, 0.}, {2.99949, 5.42517, 0.}}	{18.4302, 45.2309, 100.}	inf	27.	6.75184	{2.01312, 12.7087}	99.7381	2.68286	newly	1330.	5.05	{1., 9.}	99.6203	2.5655	Reff	0.0191979	0.10067	{0.100491, 0.100812}	424.382	ar	99 355.	96 644.6	{96 639., 96 653.}	2.72799
10	R0	{2.48791, 18.9003, 2.}	{2.73634, 13.3345, 1.}	{{2.73634, 13.3345, 1.}, {2.73634, 13.3345, 1.}}	{9.98555, 29.4484, 50.}	inf	387.	6075.52	{6017.12, 6146.89}	59.4587	0.392115	newly	4707.	2658.15	{2607., 2713.}	43.5277	0.248223	Reff	0.0381148	0.296331	{0.294362, 0.297821}	677.471	ar	99 291.	91 366.6	{91 274., 91 460.}	7.98097
11	R0	{2.84414, 18.4765, 1.}	{2.5684, 9.75224, 1.}	{{2.5684, 9.75224, 1.}, {2.5684, 9.75224, 1.}}	{9.69491, 47.2182, 0.}	inf	161.	4295.43	{4242.81, 4342.03}	67.8293	0.492558	newly	4255.	2383.5	{2333., 2436.}	43.9835	0.251752	Reff	0.0214164	0.256084	{0.254855, 0.257358}	1095.74	ar	99 654.	91 367.1	{91 290., 91 448.}	8.31563
12	R0	{2.29369, 14.622, 0.1}	{2.91946, 11.8546, 2.}	{{2.91946, 11.8546, 2.}, {2.91946, 11.8546, 2.}}	{27.282, 18.9261, 1900.}	inf	189.	3174.66	{3146.86, 3203.68}	59.7839	0.395612	newly	3139.	1485.	{1447., 1523.}	52.6919	0.325145	Reff	0.0347723	0.187556	{0.186681, 0.188342}	439.382	ar	98 982.	94 259.1	{94 202., 94 307.}	4.77143
13	R0	{2.73413, 16.7529, 1.}	{2.80387, 10.6536, 0.}	{{2.80387, 10.6536, 0.}, {2.80387, 10.6536, 0.}}	{2.5506, 36.4074, 100.}	inf	128.	3289.67	{3244.54, 3331.61}	68.0087	0.494989	newly	3594.	1688.69	{1643., 1737.}	53.0136	0.328143	Reff	0.0212989	0.221515	{0.22033, 0.222722}	940.031	ar	99 543.	92 968.2	{92 902., 93 032.}	6.60495
14	R0	{1.79554, 16.5943, 2.}	{2.93978, 19.9817, 0.}	{{2.93978, 19.9817, 0.}, {2.93978, 19.9817, 0.}}	{63.7265, 20.4132, 100.}	inf	1182.	12 317.2	{12 268.2, 12 366.6}	8.98395	0.040884	newly	4850.	3783.04	{3714., 3848.}	21.9992	0.107939	Reff	0.106063	0.244142	{0.242683, 0.245563}	130.187	ar	96 821.	94 703.2	{94 627., 94 774.}	2.18729
15	R0	{1.91429, 13.7771, 1.}	{2.37615, 9.11994, 4.}	{{2.37615, 9.11994, 4.}, {2.37615, 9.11994, 4.}}	{24.1271, 33.8036, 300.}	inf	383.	1085.13	{1043.14, 1126.69}	86.5901	0.872783	newly	3380.	635.88	{601., 667.}	81.187	0.725885	Reff	0.0667896	0.258499	{0.257511, 0.259495}	287.034	ar	97 541.	89 491.4	{89 428., 89 548.}	8.25249
16	R0	{1.42094, 10.5871, 2.}	{2.5684, 13.7324, 1.}	{{2.5684, 13.7324, 1.}, {2.5684, 13.7324, 1.}}	{80.7536, 29.7091, 50.}	inf	1054.	6916.32	{6866.94, 6962.42}	6.86341	0.030886	newly	4015.	2956.59	{2904., 3009.}	26.3614	0.132935	Reff	0.134932	0.284057	{0.28286, 0.285316}	110.518	ar	93 044.	91 268.7	{91 156., 91 358.}	1.90798
17	R0	{1.45515, 6.16466, 0.1}	{2.9751, 8.95585, 1.}	{{2.9751, 8.95585, 1.}, {2.9751, 8.95585, 1.}}	{104.453, 45.2773, 900.}	inf	170.	2241.5	{2207.15, 2275.93}	29.1933	0.111211	newly	1376.	1306.31	{1267., 1343.}	5.07049	0.0226998	Reff	0.105091	0.20485	{0.203775, 0.205842}	94.9265	ar	93 254.	93 660.1	{93 607., 93 723.}	0.435445
18	R0	{2.85669, 13.2416, 0.1}	{2.97612, 8.62388, 4.}	{{2.97612, 8.62388, 4.}, {2.97612, 8.62388, 4.}}	{4.18069, 34.8728, 3900.}	inf	31.	532.987	{512.396, 554.687}	91.3095	1.06121	newly	2583.	313.84	{293., 338.}	87.8498	0.916028	Reff	0.014512	0.182704	{0.182055, 0.183311}	1158.99	ar	99 644.	93 978.7	{93 944., 94 016.}	5.68553
19	R0	{2.56417, 17.9558, 0.1}	{2.64721, 11.9965, 0.}	{{2.64721, 11.9965, 0.}, {2.64721, 11.9965, 0.}}	{3.23858, 33.1886, 100.}	inf	266.	5534.22	{5481.54, 5578.96}	59.9608	0.397523	newly	4524.	2615.42	{2564., 2669.}	42.1879	0.238036	Reff	0.0335137	0.261036	{0.25984, 0.262239}	678.893	ar	99 389.	91 859.7	{91 780., 91 933.}	7.57558
20	R0	{2.27517, 11.1864, 0.1}	{2.97612, 8.62388, 4.}	{{2.97612, 8.62388, 4.}, {2.97612, 8.62388, 4.}}	{30.8087, 22.9075, 3900.}	inf	92.	15.7904	{3.91915, 29.741}	99.6352	2.54225	newly	2013.	9.64	{2., 18.}	99.5211	2.44636	Reff	0.0313746	0.172115	{0.171693, 0.172452}	448.581	ar	98 894.	94 220.1	{94 205., 94 238.}	4.72622
21	R0	{2.35735, 10.4735, 0.1}	{2.95668, 7.22215, 1.}	{{2.95668, 7.22215, 1.}, {2.95668, 7.22215, 1.}}	{25.4239, 31.0436, 900.}	inf	65.	15.0319	{3.54973, 27.8365}	99.5959	2.5047	newly	1848.	9.8	{2., 16.}	99.4697	2.4321	Reff	0.0275103	0.14597	{0.145533, 0.146309}	430.603	ar	99 036.	95 065.8	{95 053., 95 082.}	4.00889
22	R0	{1.79551, 11.9179, 2.}	{2.99939, 13.7076, 1.}	{{2.99939, 13.7076, 1.}, {2.99939, 13.7076, 1.}}	{67.0496, 15.0172, 50.}	inf	355.	4446.97	{4410.03, 4478.33}	28.5857	0.146222	newly	2882.	1853.04	{1810., 1897.}	35.703	0.191887	Reff	0.0710663	0.182754	{0.181606, 0.183751}	157.16	ar	97 033.	94 804.6	{94 754., 94 856.}	2.29656
23	R0	{1.23545, 6.31687, 0.1}	{2.64223, 11.0005, 3.}	{{2.64223, 11.0005, 3.}, {2.64223, 11.0005, 3.}}	{113.868, 74.1443, 2900.}	inf	264.	3468.9	{3428.3, 3505.26}	109.096	0.320328	newly	1370.	1767.36	{1724., 1811.}	29.0044	0.110526	Reff	0.148353	0.276432	{0.275452, 0.277486}	86.3344	ar	88 739.	90 750.6	{90 680., 90 816.}	2.26684
24	R0	{2.24089, 10.1789, 0.1}	{2.95668, 7.22215, 1.}	{{2.95668, 7.22215, 1.}, {2.95668, 7.22215, 1.}}	{31.9422, 29.0478, 900.}	inf	88.	143.764	{111.035, 169.252}	96.5705	1.47045	newly	2182.	95.81	{74., 114.}	95.6091	1.36303	Reff	0.0325825	0.165285	{0.164401, 0.166126}	407.282	ar	98 868.	94 439.5	{94 403., 94 471.}	4.47921
25	R0	{1.57298, 10.2607, 0.1}	{2.64721, 11.9965, 0.}	{{2.64721, 11.9965, 0.}, {2.64721, 11.9965, 0.}}	{68.2928, 16.9172, 100.}	inf	633.	4148.8	{4100.57, 4195.24}	36.7465	0.19893	newly	3489.	1961.29	{1915., 2003.}	43.7865	0.250238	Reff	0.10572	0.258479	{0.257249, 0.259755}	144.494	ar	94 957.	91 553.	{91 469., 91 635.}	3.58479
26	R0	{1.2154, 8.2576, 0.1}	{2.7499, 15.3124, 0.}	{{2.7499, 15.3124, 0.}, {2.7499, 15.3124, 0.}}	{126.255, 85.4342, 100.}	inf	684.	8648.89	{8612.21, 8685.84}	123.197	0.348686	newly	2648.	3348.4	{3294., 3406.}	26.4502	0.10188	Reff	0.165416	0.261793	{0.260674, 0.262797}	58.2636	ar	88 421.	92 988.4	{92 901., 93 065.}	5.16548
27	R0	{2.22841, 18.7983, 2.}	{2.93371, 17.1903, 1.}	{{2.93371, 17.1903, 1.}, {2.93371, 17.1903, 1.}}	{31.6502, 8.55398, 50.}	inf	454.	8587.4	{8548.28, 8621.39}	33.6419	0.178109	newly	4125.	2983.31	{2933., 3030.}	27.6773	0.14076	Reff	0.0518997	0.209367	{0.208273, 0.210491}	303.407	ar	98 756.	94 744.1	{94 676., 94 812.}	4.06244
28	R0	{1.27778, 5.36767, 1.}	{2.69353, 8.5131, 2.}	{{2.69353, 8.5131, 2.}, {2.69353, 8.5131, 2.}}	{110.798, 58.5996, 100.}	inf	160.	2148.53	{2111.36, 2182.43}	93.04	0.285607	newly	965.	1324.43	{1283., 1364.}	37.2466	0.137396	Reff	0.136275	0.263682	{0.262769, 0.264652}	93.4925	ar	89 843.	90 940.2	{90 868., 91 002.}	1.22126
29	R0	{2.38042, 15.8901, 2.}	{2.8186, 12.0293, 1.}	{{2.8186, 12.0293, 1.}, {2.8186, 12.0293, 1.}}	{18.4078, 24.2972, 50.}	inf	266.	5584.76	{5538.12, 5629.35}	52.385	0.322265	newly	4278.	2616.42	{2565., 2672.}	38.8401	0.21359	Reff	0.0399673	0.228003	{0.226694, 0.229304}	470.476	ar	99 112.	93 360.5	{93 287., 93 435.}	5.80308
30	R0	{2.53188, 12.7607, 0.1}	{2.97536, 8.97519, 1.06}	{{2.9751, 8.95585, 1.}, {2.9751, 8.95585, 1.}}	{17.5158, 29.6654, 960.}	inf	140.	3127.22	{3109.42, 3188.82}	61.3828	0.413771	newly	3519.	1802.17	{1757., 1862.}	48.7874	0.291367	Reff	0.0262556	0.182921	{0.180954, 0.183387}	596.692	ar	99 317.	94 505.9	{94 485., 94 584.}	4.84417
31	R0	{1.24482, 10.5471, 1.}	{2.29055, 13.7413, 0.}	{{2.29055, 13.7413, 0.}, {2.29055, 13.7413, 0.}}	{84.0066, 30.2852, 100.}	inf	1178.	7123.91	{7074.26, 7166.36}	18.8111	0.0748505	newly	3288.	3061.42	{3004., 3109.}	6.89112	0.0310559	Reff	0.174511	0.32226	{0.321232, 0.323373}	84.664	ar	89 097.	88 797.3	{88 694., 88 912.}	0.33642
32	R0	{2.30446, 17.9607, 1.}	{2.99548, 15.5933, 2.}	{{2.99911, 15.6207, 2.}, {2.99911, 15.6207, 2.}}	{29.9863, 13.1808, 100.}	inf	386.	7830.12	{7801.28, 7895.32}	40.8735	0.228251	newly	4308.	2965.84	{2909., 3024.}	31.1551	0.162181	Reff	0.0453748	0.232675	{0.231008, 0.23377}	412.783	ar	99 028.	94 208.3	{94 122., 94 297.}	4.86697
33	R0	{2.31579, 11.8236, 2.}	{2.97612, 8.62388, 4.}	{{2.97612, 8.62388, 4.}, {2.97612, 8.62388, 4.}}	{28.5142, 27.0622, 100.}	inf	79.	359.91	{337.932, 382.19}	91.7997	1.0867	newly	2012.	211.51	{194., 230.}	89.4876	0.979277	Reff	0.0280905	0.158362	{0.157662, 0.159}	463.756	ar	99 012.	94 746.9	{94 714., 94 779.}	4.30765
34	R0	{1.6678, 10.6744, 1.}	{2.99939, 13.7076, 1.}	{{2.99939, 13.7076, 1.}, {2.99939, 13.7076, 1.}}	{79.8413, 28.4159, 0.}	inf	325.	3134.39	{3097.47, 3169.45}	33.083	0.174479	newly	2446.	1311.81	{1279., 1345.}	46.3692	0.270675	Reff	0.0850078	0.209106	{0.208043, 0.210145}	145.984	ar	95 809.	93 800.7	{93 735., 93 856.}	2.09616
35	R0	{1.65476, 7.07559, 0.1}	{2.95668, 7.22215, 1.}	{{2.95668, 7.22215, 1.}, {2.95668, 7.22215, 1.}}	{78.6773, 2.07137, 900.}	inf	106.	525.64	{502.873, 548.016}	68.8417	0.506713	newly	1151.	351.16	{332., 371.}	69.4909	0.516004	Reff	0.0754074	0.172208	{0.171531, 0.17287}	128.37	ar	95 746.	94 282.3	{94 254., 94 315.}	1.52869
36	R0	{2.71807, 18.5959, 0.1}	{2.6266, 11.0527, 1.}	{{2.6266, 11.0527, 1.}, {2.6266, 11.0527, 1.}}	{3.36532, 40.5639, 900.}	inf	279.	5714.72	{5657.98, 5763.76}	63.3343	0.43575	newly	5029.	2911.29	{2854., 2958.}	42.11	0.237444	Reff	0.0291921	0.294817	{0.293526, 0.296273}	909.921	ar	99 540.	90 847.3	{90 746., 90 948.}	8.73286
37	R0	{2.6202, 12.7552, 0.1}	{2.97612, 8.62388, 4.}	{{2.97612, 8.62388, 4.}, {2.97612, 8.62388, 4.}}	{13.5837, 32.3893, 3900.}	inf	74.	1676.07	{1635.35, 1707.77}	74.9691	0.601581	newly	2840.	989.92	{953., 1026.}	65.1437	0.457881	Reff	0.0220097	0.176601	{0.175655, 0.177801}	702.378	ar	99 426.	94 417.2	{94 364., 94 472.}	5.03775
38	R0	{1.47672, 9.81651, 1.}	{2.73634, 13.3345, 1.}	{{2.73634, 13.3345, 1.}, {2.73634, 13.3345, 1.}}	{85.2986, 35.8372, 0.}	inf	503.	5230.44	{5194.75, 5265.92}	12.169	0.0498666	newly	2643.	2239.78	{2187., 2287.}	15.2561	0.0719482	Reff	0.109425	0.201	{0.200007, 0.201908}	83.6872	ar	93 815.	93 830.6	{93 761., 93 896.}	0.0468795
39	R0	{1.90004, 12.3313, 1.}	{2.99939, 13.7076, 1.}	{{2.99939, 13.7076, 1.}, {2.99939, 13.7076, 1.}}	{57.8595, 11.1613, 0.}	inf	369.	4731.07	{4693.06, 4770.39}	35.1731	0.188253	newly	3317.	1975.26	{1926., 2019.}	40.4504	0.225199	Reff	0.0681354	0.195618	{0.194386, 0.196736}	187.102	ar	97 413.	94 514.4	{94 445., 94 584.}	2.97563
40	R0	{2.64218, 18.5237, 2.}	{2.99939, 13.7076, 1.}	{{2.99939, 13.7076, 1.}, {2.99939, 13.7076, 1.}}	{13.5196, 25.9995, 50.}	inf	174.	4932.86	{4895.38, 4969.29}	57.5814	0.372451	newly	3674.	2078.71	{2036., 2118.}	43.4211	0.247407	Reff	0.0267917	0.236476	{0.235356, 0.237584}	782.648	ar	99 474.	93 470.3	{93 410., 93 542.}	6.03542
41	R0	{1.6945, 7.17996, 0.1}	{2.95668, 7.22215, 1.}	{{2.95668, 7.22215, 1.}, {2.95668, 7.22215, 1.}}	{74.4869, 0.587633, 900.}	inf	141.	624.686	{596.746, 655.051}	74.9122	0.600842	newly	1635.	416.3	{395., 441.}	74.5382	0.594566	Reff	0.0694067	0.180426	{0.179498, 0.181246}	159.954	ar	96 332.	94 031.5	{93 993., 94 070.}	2.38808
42	R0	{1.84559, 18.0074, 1.}	{2.96978, 18.8857, 0.}	{{2.96978, 18.8857, 0.}, {2.96978, 18.8857, 0.}}	{60.9123, 4.87761, 100.}	inf	1048.	10 540.4	{10 498.4, 10 592.2}	22.9447	0.1132	newly	4650.	3420.39	{3356., 3489.}	26.4432	0.133424	Reff	0.093313	0.274485	{0.272945, 0.27572}	194.155	ar	97 358.	93 829.1	{93 741., 93 908.}	3.62466
43	R0	{2.24965, 17.0361, 2.}	{2.73634, 13.3345, 1.}	{{2.73634, 13.3345, 1.}, {2.73634, 13.3345, 1.}}	{21.6341, 21.7281, 50.}	inf	520.	5823.59	{5767.22, 5873.2}	56.7019	0.363541	newly	4598.	2545.19	{2501., 2597.}	44.6457	0.256898	Reff	0.0537891	0.288339	{0.286928, 0.289705}	436.055	ar	98 770.	91 495.7	{91 401., 91 597.}	7.36485
44	R0	{2.54324, 15.7967, 0.1}	{2.80387, 10.6536, 0.}	{{2.80387, 10.6536, 0.}, {2.80387, 10.6536, 0.}}	{10.2478, 32.558, 100.}	inf	127.	2686.93	{2649.25, 2721.68}	69.7247	0.518934	newly	3246.	1371.11	{1331., 1408.}	57.76	0.374391	Reff	0.0255087	0.205189	{0.204208, 0.206235}	704.388	ar	99 354.	93 344.1	{93 290., 93 404.}	6.04893
45	R0	{2.54943, 14.8032, 0.1}	{2.93906, 10.3908, 0.}	{{2.93906, 10.3908, 0.}, {2.93906, 10.3908, 0.}}	{15.2829, 29.8072, 100.}	inf	120.	3079.75	{3043.36, 3119.23}	62.2441	0.423033	newly	3187.	1591.26	{1555., 1633.}	50.0703	0.301725	Reff	0.0250609	0.181641	{0.18047, 0.18269}	624.799	ar	99 380.	94 456.3	{94 397., 94 507.}	4.95443
46	R0	{2.37755, 12.6461, 2.}	{2.98388, 9.60029, 3.}	{{2.98388, 9.60029, 3.}, {2.98388, 9.60029, 3.}}	{25.5023, 24.085, 50.}	inf	129.	2661.62	{2623.21, 2700.09}	63.2372	0.43462	newly	3124.	1457.47	{1413., 1493.}	53.346	0.331203	Reff	0.0309795	0.179342	{0.178068, 0.180423}	478.907	ar	99 096.	94 537.6	{94 479., 94 593.}	4.6
47	R0	{1.69279, 11.6636, 1.}	{2.53075, 11.6597, 0.}	{{2.53075, 11.6597, 0.}, {2.53075, 11.6597, 0.}}	{49.5017, 0.033314, 100.}	inf	535.	2563.81	{2522.53, 2606.23}	65.7061	0.464815	newly	3657.	1242.89	{1213., 1277.}	66.0134	0.46878	Reff	0.0899041	0.272263	{0.271151, 0.273292}	202.837	ar	96 214.	90 142.4	{90 066., 90 216.}	6.31047
48	R0	{1.82402, 10.0038, 1.}	{2.93906, 10.3908, 0.}	{{2.93906, 10.3908, 0.}, {2.93906, 10.3908, 0.}}	{61.1307, 3.86835, 100.}	inf	312.	2705.47	{2668.86, 2738.34}	48.2503	0.286118	newly	2763.	1402.85	{1357., 1442.}	49.2273	0.294486	Reff	0.0676164	0.192259	{0.191233, 0.193324}	184.338	ar	97 076.	94 063.7	{94 007., 94 117.}	3.10303
49	R0	{1.6424, 11.1667, 2.}	{2.86001, 13.5542, 0.}	{{2.86001, 13.5542, 0.}, {2.86001, 13.5542, 0.}}	{74.1359, 21.3808, 100.}	inf	368.	2641.62	{2608.57, 2679.67}	45.0578	0.260114	newly	2454.	1123.09	{1086., 1162.}	54.2343	0.33959	Reff	0.0891166	0.227667	{0.226554, 0.228497}	155.471	ar	95 546.	92 766.6	{92 711., 92 826.}	2.90901
50	R0	{2.05638, 14.3048, 1.}	{2.99939, 13.7076, 1.}	{{2.99939, 13.7076, 1.}, {2.99939, 13.7076, 1.}}	{45.8579, 4.17462, 0.}	inf	362.	5649.6	{5605.31, 5687.26}	38.9629	0.214412	newly	3752.	2365.9	{2317., 2417.}	36.943	0.200333	Reff	0.0564271	0.201331	{0.200187, 0.202613}	256.799	ar	98 194.	94 543.9	{94 468., 94 605.}	3.71727
